# Have We a Responsible Government?

**Published:** 1911-11-25

**Authors:** 


### The Hospital

The Workers' Newspaper of Administrative Medicine and Institutional Life, Administration, National Insurance and Health.

No. 1324, Vol. LI. SATURDAY, NOVEMBER 25TH, 1911. PRICE ONE PENNY.

### HAVE WE A RESPONSIBLE GOVERNMENT?

Mr. Asquith announced on November 20 that the Report stage of the National Insurance Bill would be taken on Tuesday, November 28. At the present time the Bill makes no provision for institutional or hospital benefit, whilst it recruits at least 400,000 persons who will require in-patient hospital treatment every year without providing one single bed for their accommodation. The Bill as it stands, as we have already shown, must paralyse and may ultimately destroy the voluntary hospitals. In any case the 400,000 in-patients recruited by the Government under the National Insurance Bill will be without hospital accommodation when seriously ill, to the risk of their health and even of their lives.

Such is the position of the Government in regard to severe illness in the case of all persons insured under Mr. Lloyd George's Bill. Mr. Lloyd George has claimed that "he will provide adequate medical treatment for every workman in the Kingdom," and that this relief is to be free from any taint of charity. But, whatever his intentions, his Bill as it stands directly confutes these claims, for it makes no such provision and must indeed destroy much of the charitable hospital provision already in existence. We desire to recognise fully the stress and strain under which Mr. Lloyd George has had to labour, but that strain and stress are due almost entirely to the fact that neither sufficient time nor adequate knowledge has been brought to bear upon the construction of this scheme of national insurance. The natural consequence is that at present there is nobody living, probably, who can give a reasonable guess as to exactly how any clause of the Bill will operate should it became law, much less what the effects of this Bill will be upon the national and charitable revenues, upon the medical profession, or upon the lives, health, and happiness of the people, or even upon the Government and Parliament which could have the temerity to pass it in its present form.

What are the managers of voluntary hospitals doing in defence of the poor who are grievously ill and of the insured who must suffer grave injury in similar circumstances, unless the Bill is amended by the inclusion of hospital and institutional benefit? It is a unique position that the Government of the day should tell the country that they are doing an immense national service by "securing the workers of the country against the distress which is incidental to the illness of a breadwinner," when the proposed legislation is found on examination to be so faulty, as to render it essential for the representatives of the voluntary hospitals throughout the United Kingdom to combine, to protect the unfortunate sick from the injury which must be inflicted upon them under this very Bill, unless several of its present clauses are amended before it becomes law. So unanimous is the conviction that this course is essential that practically the whole of the great voluntary hospitals throughout the country have resolved to send one, two, and in some cases three representatives to a great meeting of hospital managers, which will be held at the Westminster Palace Hotel, London, on the 1st proximo. It is now certain that this meeting will prove to be the most representative gathering of hospital managers that has ever been held in this country. The members of the British Medical Association are in agreement with the hospital managers in resolving that it is essential again to press the Government to make adequate provision in the Insurance Bill for institutional, that is for hospital treatment. Without it the National Insurance Bill must prove in practice to be an injury and a grievous wrong to tens of thousands of poor people when seriously ill. With it, it should be possible so to rearrange our whole system of health and hospital provision for all classes in this country as to make it not only the most efficient, but the least costly of organisations of the kind which the world has yet known.

We wish we could express some confidence that time will be found to discuss this important question of hospital benefit in the House of Commons before the Bill is forced through. Judged by the past, there would seem to be little hope that we can secure a fair discussion of the claims of the great army of sick people in these islands, who must suffer many avoidable injuries and dangers, unless hospital benefit is included in the Bill. It is a grievous thing to have to print, but it is true, that the interests of the voluntary hospitals have been entirely sacrificed, so far, in the House of Commons, owing to the rush and hurry with which the Insurance Bill has been handled. We understood Mr. Lloyd George to promise definitely that the just claims of the voluntary hospitals to consideration and protection should have full consideration when the Bill was in Committee. So far this pledge is unfulfilled.

The outlook for the voluntary hospitals, bad as it is, is not nearly as bad, nor half as cruel, as the lot of the insured when seriously ill must prove to be under the National Insurance Bill as it stands on emerging from Committee. The chances of effecting amendments in the Bill, as the result of the great meeting in London, must be minimised by the circumstance that, it is not to be held until December 1. Whatever the result, we make bold to say that, somehow or other, failing the inclusion of institutional or hospital benefit in the Bill, steps should be taken, quite apart from politics, to render it plain to the whole nation that when passed it will inflict more avoidable suffering on the workmen when ill than they have ever before had to face.

